# Outcome of crisis intervention for borderline personality disorder and post traumatic stress disorder: a model for modification of the mechanism of disorder in complex post traumatic syndromes

**DOI:** 10.1186/1744-859X-9-19

**Published:** 2010-04-27

**Authors:** Andreas Laddis

**Affiliations:** 1 Riverside Community Care, Bellingham, MA, USA; 2 School of Public Health of the Boston University, Boston, MA, USA; 3 The International Society for the Study of Trauma and Dissociation, McLean, VA, USA

## Abstract

**Background:**

This study investigates the outcome of crisis intervention for chronic post traumatic disorders with a model based on the theory that such crises manifest trauma in the present. The sufferer's behavior is in response to the current perception of dependency and entrapment in a mistrusted relationship. The mechanism of disorder is the sufferer's activity, which aims to either prove or disprove the perception of entrapment, but, instead, elicits more semblances of it in a circular manner. Patients have reasons to keep such activity private from therapy and are barely aware of it as the source of their symptoms.

**Methods:**

The hypothesis is that the experimental intervention will reduce symptoms broadly within 8 to 24 h from initiation of treatment, compared to treatment as usual. The experimental intervention sidesteps other symptoms to engage patients in testing the trustworthiness of the troubled relationship with closure, thus ending the circularity of their own ways. The study compares 32 experimental subjects with 26 controls at similar crisis stabilization units.

**Results:**

The results of the Brief Psychiatric Rating Scale (BPRS) supported the hypothesis (both in total score and for four of five subscales), as did results with Client Observation, a pilot instrument designed specifically for the circular behavior targeted by the experimental intervention. Results were mostly non-significant from two instruments of patient self-observation, which provided retrospective pretreatment scores.

**Conclusions:**

The discussion envisions further steps to ascertain that this broad reduction of symptoms ensues from the singular correction that distinguishes the experimental intervention.

**Trial registration:**

Protocol Registration System NCT00269139. The PRS URL is https://register.clinicaltrials.gov

## Background

Behavioral crises in the course of borderline personality disorder (BPD) and post traumatic stress disorder (PTSD) consist of intrusive rehearsals of old entrapment in danger, dissociative states with unstoppable irrational urges, hallucinations, mood lability and impulsivity. They are notoriously costly in utilization of acute services [1-5]. This study investigates a clinical intervention that may offer quick reduction of symptoms to reduce those costs. The experimental intervention is part of the Cape Cod Model of psychotherapy [6].

### Behavioral crises in chronic post traumatic disorders

There is a domain of study that aspires to demonstrate that complex PTSD and BPD are related. These studies attribute to both a hypothesized post traumatic mechanism of disorder resulting from dependency in a relationship with mistrusted caretakers, individuals or institutions [7,8]. Those caretakers controlled the dependent's means to ascertain and correct the caretakers' trustworthiness as well as the dependent's means to leave the relationship. Differently from simple PTSD, survivors of that particular trauma recreate semblances of dependency in later relationships, semblances of others' betrayal and of their own powerlessness.

The hypothesized shared mechanism of disorder for complex PTSD and BPD has not been investigated empirically. Still, their similarity in personality development and the phenomenology of their crises is evident. Guilt, shame, loss of faith in the benevolence of others, hopelessness, mistrust and avoidance of primary relationships are personality attributes of persons with complex PTSD [9-16], a 'unique trademark' that distinguishes it from simple PTSD [17]. As these attributes were found also in BPD, some authors subsumed them in concepts of post traumatic personality disorder [15,18]. The description resembles the diagnostic category of the *International Classification of Diseases*, 10th edition (ICD-10) [19] called 'enduring personality changes after catastrophic experience', such as lengthy captivity in adult life.

The crises of both complex PTSD and BPD are characterized by the sufferer's instigation of others to behave in ways that resemble entrapment by mistrusted caretakers [20]. That activity is commonly recognized in the clinical literature as 'repetition compulsion', with various explanations [21-27]. In complex PTSD and BPD, the classic symptoms of post traumatic disorder, vigilance, numbness and flashbacks, happen in the course of repetition compulsion. For the purposes of this study, the term 'behavioral crisis' is used only for this complex presentation. A typical description of it is given in the next paragraph, as it was provided to clinicians for recognition of prospective subjects, before diagnostic screening.

Typical behavioral crises are a composite of many unresolved semblances of dependency in mistrusted relationships from one crisis to the next. The person's judgments about blame for the entrapment become ever more uncertain. For example, a man who hears hallucinatory voices saying 'you are a loser' cannot be sure if that judgment was inflicted on him by his father who used to lock him in the closet or by his mother who never brought him the food and water that she promised. The voice sometimes sounds like an admired teacher's whose class he never dared attend. His recollection shifts with endless doubts about who wanted him in the role of loser, including himself. Sometimes he doubts the factuality of a particular event altogether. The means of testing others' commitment grow ever stranger and costlier, in terms of sacrifices, demands and acts of atonement. He self-mutilates, binges on food or sex, menaces for trivial wants and against trivial dangers. The force and repetitiveness of these activities blind him to his own intervening needs and to others' feelings and reasons. Afterwards, he remembers all that blundering very inexactly.

### Efficacy of treatment

Studies of the efficacy of treatments for behavioral crises are reviewed here in aggregate, for both BPD and PTSD. The distinction between simple and complex PTSD had not been made yet at the time of these studies and reviews.

Studies of outcomes with long-term pharmacotherapy for these disorders pertain mainly to mitigation of behavioral crises (for example, of irrational and shifting moods, impulsivity and psychotic symptoms). In summary, reviews of those studies find the evidence sparse and inconclusive, with trends in support of modest improvement of each symptom for selected drugs [28-40]. Professional practice guidelines emphasize the symptomatic nature of relief with medication [36,41-43]. As such, medication is a useful adjunct to psychotherapy that, in turn, may repair the mechanism of crises, thereby making medication unnecessary. Some authors explain the limitations of pharmacotherapy by the nature of BPD and complex PTSD as disorders of social learning [30,44].

For long-term psychotherapy as well, studies of outcomes with particular schools [45-51], reviews of studies [33,52-64] and practice guidelines [36,42,43] agree that crises become fewer, with less acting out and intensity. Patients consistently become less angry, labile and impulsive; they self-mutilate less and make fewer suicide gestures.

In recognition of how difficult it is to engage patients in new insights during crises, much of psychotherapy in the intervals promotes the value of self-policing, self-soothing and welcoming others' help with the same. Nonetheless, several programs had similar results with an abbreviated, intense course of various psychotherapies, tailored for crisis times [65-74].

### Reparative and symptomatic psychotherapy

Beyond reviewing the efficacy of long-term psychotherapy for reduction of crises, the theory underlying the experimental intervention makes it relevant to review the efficacy also for deep structural reparation of the mechanism of disorder. The theory of the Cape Cod Model claims that the experimental intervention achieves reparation of that mechanism, in measurable increments from one application to the next. This study introduces pilot instruments to begin measuring the patient's experience of modification of the putative mechanism.

Remarkably, the efficacy for reduction of behavioral crises cited in the preceding section is similar among the different schools of psychotherapy [47,58,75]. For the early stages of therapy, the different schools borrow among them short-term techniques that mitigate burdensome symptoms. By design, all therapies included in these reviews advise patients to forego expectations for intimacy in unfulfilled old relationships or in new ones until after in-therapy lessons accumulate. They all promise gradual deep correction of the patients' response to danger in intimate relationships, though via sharply different interventions. So far, the evidence suggests success from the techniques that the different schools share in early phases. Results for later phases, however, which they each promise to obtain differently, have not been demonstrated yet. Outcome studies show consistently that patients become more compliant with treatment, mingle with others more comfortably and take better care of themselves [24,47,61,62]. However, the evidence is less consistent for improvement of symptoms in the intervals between behavioral crises, that is, anxiety and depression, dysphoria, paranoia and dysfunctional beliefs [24,47,58,61,62]. There is no significant improvement for a residual cluster of symptoms, a 'subsyndrome' [62] of hopelessness, emptiness and fear of intimacy.

With these concerns in mind, Benjamin and Linehan proposed to measure therapy's efficacy in degrees of reparation of the 'core dysfunction' in complex post traumatic syndromes [76-78]. Reparation should show as competence in intimate relationships, having 'a life worth living', beyond the passage 'from loud to quiet desperation'. They envisioned a research program that will identify the true core dysfunction as hypothesized by competing theories and measure its gradual correction. Otherwise 'what is a "symptom" to one [author] may be the mechanism controlling a disorder to another' [76]. Benjamin nominates 'underlying destructive attachments' as the core dysfunction to investigate. A concept akin to Benjamin's, that of regressive social learning, guides the Cape Cod Model of treatment during and between crises [20].

### The Cape Cod Model

According to the Cape Cod Model, the irrational and unstoppable activity of behavioral crises is the sufferer's way of coping with perceived entrapment in a current treacherous relationship. The entrapment, whether true or false, consists of the perception of betrayal which the person cannot ascertain one way or the other. The sufferer can neither become certain enough of the other's trustworthiness to recommit to the current relationship, nor can he become certain enough to move on, confident to ascertain betrayal in later relationships.

The mechanism of disorder is in the sufferers' regressive method of testing the other's fidelity to promises and expectations, commonly recognized as repetition compulsion [20]. Regressive testing elicits more semblances of betrayal, which compounds their sense of their own entrapment. Each round of testing renders them more uncertain than before. This circular, self-defeating activity replicates the method that survivors of dependency in mistrusted relationships learned as the way to test their caretakers' trustworthiness.

The Cape Cod Model explains the course of chronic post traumatic disorder over the lifetime in terms of a social breakdown syndrome. Cumulatively, from one crisis to the next, survivors of entrapment in failed caretaking relationships mislearn that love is indecipherable and, therefore, a dangerous gamble. They grow simultaneously more desperate for intimacy and more apprehensive of it. In response to the survivors' wasteful, repetitive testing, others also become tentative about offering opportunities for intimacy to them. The social breakdown often takes hold despite psychotherapy because patients have reasons to keep their regressive experiments private from their therapist and they are hardly aware of them as the source of behavioral crises. To observers, crises appear to emerge in response to incidental reminders of old trauma, even trivial ones. Over time, patients mislearn from their private experiments faster than they make progress in therapy with analysis of the transference and of scenarios of old betrayals.

The crisis intervention of the Cape Cod Model aims for quick resolution by offering immediate, rudimentary proof that trustworthiness is testable, directly in the troubled relationship or in an opportune relationship beyond this loss. Clinicians propose ways to make intimacy safe, ways which patients cannot envision on their own, to replace repetition compulsion, the mechanism of disorder and the source of all symptoms. From resolution of one crisis to the next, the experimental intervention cumulatively improves the sufferer's vulnerability in future relationships.

Outside crises, psychotherapy with the Cape Cod Model is designed to anticipate crises and abort the social breakdown syndrome. From the beginning of therapy, clinicians join patients in seizing opportunities for increments of intimacy in life-defining relationships. The patients' goal is to test others' trustworthiness effectively, in order to let go of repetition compulsion.

## Methods

The study was approved by the institutional review board of the Massachusetts Department of Mental Health. It was registered prospectively with the Protocol Registration System of the National Institutes of Health.

### Hypothesis

The hypothesis for this study is that all symptoms of behavioral disorder will show greater improvement with the experimental intervention than with treatment as usual within 8 to 24 h from initiation of treatment.

### Participants and recruitment

A total of 58 participants were recruited for this study. The subjects for the experimental group (n = 32) were recruited from consecutive admissions to one Crisis Stabilization Unit (CSU) and the control subjects (n = 26) were recruited at two other CSUs. The referring agency for each CSU is the service that triages behavioral emergencies for the Department of Mental Health in the same region. Patients who are found likely to become dangerous are admitted to a CSU, instead of an inpatient unit, if they appear eager for help to end the dangerousness. All three programs are unlocked residential units for stay from 1 day to weeks and serve regional agencies of the Massachusetts Department of Mental Health. They have the same mission and similar staffing, for both psychosocial and pharmacological interventions. They serve similar populations demographically, in terms of educational and socioeconomic status and access to treatment between crises. Table 1 provides a description of the demographic characteristics of the control and experimental groups. There was a significant difference in gender between the two groups but not in age, marital status or education.

**Table 1:** Demographic characteristics

	Control group (n = 26)	Experimental group (n = 32)	Significance
Gender:			*P *= 0.03
Male	1	8	
Female	25	24	
Age (mean)	33.2	37.2	NS
Marital status:			NS
Single	18	24	
Divorced	7	4	
Married	1	4	
Education:			NS
< High school	7	10	
High school and General Educational Development (GED)	10	17	
≥ 1 year college	9	5	

NS = not significant.

Subjects were judged by licensed master's level clinicians to be dangerous to themselves or others on account of behavioral crisis, as described in the Introduction. Those patients were approached for informed consent to participate in the study 8 to 24 h from initiation of treatment. If they accepted, they were screened for BPD (n = 54) or PTSD (n = 4) by structured interview. Clients were ineligible for the study if there was evidence of brain damage or current intoxication or withdrawal from addictive substances. All clients approached for recruitment accepted, and of those who met the diagnostic criteria all but one in each group completed the study.

### Measures

The Structured Clinical Interview for Diagnostic and Statistical Manual of Mental Disorders, 4th edition (DSM-IV) Axis I Disorders, Clinical Version (SCID-I) and the Structured Clinical Interview for DSM-IV Personality Disorders (SIDP-IV) were used for diagnostic screening for PTSD and BPD, respectively.

### Brief Psychiatric Rating Scale (BPRS)

The BPRS consists of 18 items and 5 subscales. The items are rated from 1 to 7 by observation and interview, according to rating instructions. For the purpose of data analysis, the scores were converted to a 0 to 6 scale so that absence of a symptom would equal a zero score. For both the experimental and the control subjects the BPRS was administered upon admission to CSU, before treatment, by master's level clinicians of a separate service who assessed and triaged psychiatric emergencies. These preadmission raters achieved inter-rater reliability (mean intraclass correlation coefficient (ICC) = 0.97 range 0.831 to 0.995) for item and total BPRS scores with the raters who administered the rest of the protocol after treatment.

### Brief Symptom Inventory (BSI)

This self-administered questionnaire consists of 53 items and 9 subscales. The ratings are from 0 (not at all) to 4 (extremely). After treatment, subjects rated their current symptoms and retrospectively rated their symptoms prior to treatment.

### Client Observation

This is a pilot rating scale developed by the author (AL). It consists of five items of observable behavior that are characteristic of behavioral crises in BPD and PTSD (see Table 2). They are outward manifestations of the underlying scenario of repetition compulsion, self-entrapment and dissociation (for example, testing others with shifting demands, reliving old submission to exploitation and entrancement). The five items were given ratings of 0 (none) to 5 (constant). A registered nurse completed ratings before and after treatment with guidance from the research staff about the criteria for each rating. The nurses' judgment was based on review of the medical record, as a summary of all staff accounts. Although the nurses assigned both ratings after treatment, their judgment about pretreatment behavior was based on a summary of notes from before treatment, their own and of other staff.

**Table 2:** Client observation total and item scores (mean (SD))

	**Experimental group (N = 32)**		**Control group (N = 26)**	
	
	**Baseline**	**Follow-up**	**Baseline**	**Follow-up**
	
Total Client Observation	19.7 (4.2)	7 (4.8)**	12.8 (3.6)	9.0 (3.2)
Repetitively self-defeating behavior	4.8 (.4)	1.7 (1.3)*	3.6 (1.1)	2.3 (1.3)
Self-absorbed or entranced	2.0 (1.3)	1.3 (1.8)**	2.1 (1.6)	1.7 (1.5)
Misperceptions of reality	2.6 (2.3)	0.7 (1.1)	0.5 (1.2)	0.4 (1.0)
Ever shifting priorities	3.8 (1.8)	1.3 (1.3)**	3.4 (1.0)	2.3 (0.9)
Is needy, with ever shifting wants	3.9 (1.8)	1.4 (1.3)**	3.3 (1.3)	2.3 (1.2)

*P ≤ 0.05; **P ≤ 0.001.

### Client Self-Observation

This pilot rating scale, developed by the author (AL), consists of nine items concerning mental events underlying the observable behavior of Client Observation (see Table 3). It is meant to tap by interview the parts of mental operations that comprise the unspoken scenario of behavioral crisis. Some parts are unique to post traumatic disorder and expected to be found in every instance of it (for example, intrusive memories and wallowing in uncertainty about ever knowing a loved one's trustworthiness); other parts, such as mental overload and shifting priorities, are characteristic of any entrapment in danger, and not exclusively post traumatic. A structured interview with research staff provided well differentiated markers for the client's self-ratings from 0 (none) to 5 (constant). It took place after treatment and included both a retrospective pretreatment and a follow-up rating.

**Table 3:** Client self-observation total and item scores (mean (SD))

	**Experimental group (N = 32)**		**Control group (N = 26)**	
	
	**Baseline**	**Follow-up**	**Baseline**	**Follow-up**
	
Total client self-observation	32.3 (6.8)	19.3 (6.8)*	35.7 (6.2)	24.7 (5.0)
Mentally overloaded, overwhelmed	4.5 (1.1)	2.3 (1.3)*	4.5 (0.9)	3.2 (1.2)
Vigilance	3.7 (1.5)	2.0 (1.3)	4.3 (1.1)	2.7 (1.2)
Circular rumination	4.3 (1.4)	2.7 (1.5)*	4.1 (1.5)	3.0 (1.3)
Helplessness and depression	4.5 (1.1)	3.0 (1.1)	4.4 (1.1)	3.1 (1.1)
Irrational urges	3.7 (1.9)	1.9 (1.6)	4.0 (1.0)	2.3 (1.1)
Intrusive flashbacks	3.1 (2.1)	2.2 (1.8)	3.7 (1.5)	2.5 (1.3)
Dissociative symptoms	1.7 (2.1)	0.8 (1.3)	2.6 (1.8)	1.6 (1.2)
Inability to make judgments of priorities	3.5 (1.9)	2.1 (1.6)	4.3 (0.9)	3.0 (1.1)
Inability to make judgments of trust	3.4 (1.9)	2.4 (1.8)	3.8 (1.0)	3.3 (1.2)

*P ≤ 0.05.

Finally, the research staff obtained a list of medications before and after treatment in order to ascertain if differences in prescribing patterns between the two groups might account for the results in the experimental condition.

### Procedure

Prospective subjects for both conditions were given the BPRS prior to their admission to the three CSUs. After admission, prospective subjects for the experimental group were treated with the crisis intervention according to the Cape Cod Model. They were offered all methods of symptom containment and diversion at first (for example, medication, grounding, relaxation, and so on) in order to lessen the force of their absorption and make the therapist's voice heard. The experimental subjects were allowed to continue or to modify their long-term medication regimen as they chose, after advice about realistic expectations from it. The reason was to avoid contamination of the results by a negative placebo effect from refusing to prescribe drugs for which, in the prescriber's opinion, patients had a superstitious preference. The subjects of the control group were given treatment as usual, consisting of medication, supportive psychotherapy, problem solving, occasional analysis of the transference and elements of Dialectical Behavioral Therapy.

In both conditions, recruitment, informed consent and testing were initiated and completed between 8 and 24 h from the beginning of treatment (that is, from the subject's examination by a psychiatrist and formulation of a treatment plan by the clinical team). Research assistants ('raters'), who were master's level clinicians from outside the CSUs, a different contingent for each CSU, implemented that entire post-treatment procedure. The variation from 8 to 24 h was for administrative reasons, such as when raters were available and did not interfere with the subjects' other commitments.

All raters had undergone the same training and testing for inter-rater reliability. The raters explained the procedure and human rights to the prospective subjects and obtained informed consent. Then they administered the structured diagnostic interviews according to the DSM-IV. For the qualified subjects, the raters administered the various measures and then interviewed the staff. Finally, they obtained the medication regimen of each subject for before and after admission.

Raters, subjects and clinical staff at all three sites were informed about the general purpose of the study, namely to compare the intervention to treatment as usual. Raters at all sites were blind to the hypothesis and to the technique used. Furthermore, clinical staff at the two control sites were blinded to the experimental hypothesis and technique, so as not to become tempted to improvise and contaminate their treatment as usual. Raters knew the designation of each site as experimental or control.

### Intervention

The object of the present study is the first phase of the intervention. The complete intervention takes place for 1 to 2 h initially and then in several shorter sessions over a period of 1 or 2 days. Every patient in behavioral crisis has a latent story of a current relationship with an object of need and fear and the therapist's first purpose is to elicit that story. Typically, the patient is loudly preoccupied with desire, mistrust, worthlessness and powerlessness in various relationships, including trivial or hallucinated ones. The therapist stimulates that preoccupation in hope of eliciting tangential associations to the relationship that matters. In the earlier example of the man who heard a voice judging him, the therapist nudges him along, 'Who thinks you are a loser...you don't know who hurt you more, your father or your mother...locked in the closet... who treats you like a loser today?'. With that nudging, the patient gropes around 'Nobody...who cares what my mother thinks...I saw her at the market yesterday, from behind the shelves...she must have seen my car outside...every day I go...who cares!'. The therapist recognizes the mother as the object of rising need and fear and speaks to that with empathy and a hint of hope, for example, 'That is no way to live!'. The patient responds with a sudden lull in his unstoppable, irrational activity. In that lull, the therapist proposes that there is indeed a better method to become sure of the mother's intentions, one way or the other, and of others' in the future.

Engagement in that proposition replaces the patient's frantic regressive testing and symptoms cease for the duration of that engagement. Over the course of the next 1 or 2 days, the patient typically breaks off and then reestablishes this therapeutic engagement, whereby symptoms resurge and cease again. Patients break the engagement because of good or bad, real or perceived developments in the troubled relationship that seduces them to make private judgments of trust again. Modulation of particular symptoms with medication, grounding, and so on, is useful to facilitate engagement and reengagement in the therapeutic proposition, but such measures become unnecessary for hours at a time, when the engagement is in effect.

### Statistical analysis plan

The statistical analysis plan was developed to test the hypothesis for greater reduction of symptoms in the experimental group than the control group. Analysis for between-group differences was performed for education and marital status using χ^2^, gender using Fisher's exact test, and age using the t test. A correlation matrix was performed to examine for any associations between the demographic variables and the total score of the BPRS, BSI, and Client Observation Scale. General linear model (mixed model analysis of variance (ANOVA)) was used to examine both within and between group differences in total BPRS, total BSI and total Client Observation scores at pretreatment and at follow-up. There was a significant difference between the two treatment groups at baseline on the pre-BPRS total score (*P *= 0.002) and gender (*P *= 0.027) therefore they were used as covariates in the analysis. Correlations for the BSI total showed a significant difference at baseline for gender (*P *= 0.027) between the two treatment groups, and this was used as a covariate for the BSI analysis. The staff Client Observation total correlations found a significant difference (*P *= 0.000) in prescores and gender (*P *= 0.027) between the two treatment groups and they were used as covariates in the analysis.

A hierarchical regression was performed to investigate the contribution of the variables to the variance in the total BPRS follow-up score (the dependent variable). A correlation matrix to examine for any associations between the independent variables found marital status and education to be highly correlated (r = 0.369, *P *= 0.003). Therefore, in the regression the independent variables were entered in four blocks with gender, age and education in block 1, marital status in block 2, pretreatment BPRS total score in block 3 and the two treatment groups (control and experimental) in block 4.

## Results

### BPRS

There was no significant difference in education, marital status, and age between the two treatment groups (see Table 1 for demographic characteristics for the two groups). There were significantly more females than males (*P *= 0.03) in both treatment groups. The general linear model for within and between group differences (control versus experimental) found a significant difference in prescores in the total BPRS score. Box's test of equality of the covariance matrices and Mauchly's test of sphericity were not significant, therefore assumptions were met. The mixed model ANOVA revealed that the main effect found significantly greater improvement in the follow-up BPRS total score for the experimental group (*M *= 12.9) than the control group (*M *= 24.7) taking into account the covariates gender and Pre-BPRS score F = 29.23, *P *< 0.001, partial Eta^2 ^= 0.35.

A hierarchical regression analysis was used to determine the effect of independent variables on the variance in the BPRS total score. Independent variables were entered into the equation in four blocks as detailed in the Methods section. In the final model neither the demographic characteristics of gender, education, age (R^2 ^= 0.037, F change = 0.693, *P *= 0.560) and marital status (R^2 ^= 0.044, F change = 0.364, *P *= 0.549) nor the pre-BPRS score (R^2 ^= 0.077, F change = 1.882, *P *= 0.176) contributed significantly to the change in the BPRS follow-up score. Only the group (control versus experimental) (R^2 ^= 0.402, F change = 27.70, *P *≤ 0.001) made a significant contribution toward the change in the BPRS follow-up score, accounting for 33% of the variance.

Since there was significant improvement in the total BPRS score for the experimental group, each of the subscales (thought disorder, withdrawal/retardation, anxiety/depression, hostility/suspiciousness, and activation) were examined to look for which symptom areas improved the most using the general linear model with the presubscale score and gender as covariates (see Table 4). Box's test of equality of the covariance matrices and Mauchly's test of sphericity were not significant, therefore, assumptions were met except Box's M was significant (*P *≤ 0.001) for the thought disorder subscale. The thought disorder prescore for the experimental group (*M *= 4.4) was significantly higher than the control group (*M *= 1.7), although there was no significant difference at follow-up between the two treatment groups (F = 3.05, *P *= 0.086, partial Eta^2 ^= 0.053). All other subscales had significant improvement in the experimental group at follow-up. (Withdrawal/retardation (F = 13.04, *P *= 0.001, partial Eta^2 ^= 0.195), anxiety/depression (F = 22.00, *P *≤ 0.001, partial Eta^2 ^= 0.289), hostility/suspiciousness (F = 17.51, *P ≤* 0.001, partial Eta^2 ^= 0.245), and activation (F = 4.83, *P *= 0.032, partial Eta^2 ^= 0.082).) The decreased scores in the anxiety/depression, hostility/suspiciousness and withdrawal/retardation subscales showed the largest effect sizes suggesting these three areas contributed the most to the change in BPRS scores.

**Table 4:** Brief Psychiatric Rating Scale (BPRS) total and subscale scores (mean (SD))

	**Experimental group (N = 32)**		**Control group (N = 26)**	
	
	**Baseline**	**Follow-up**	**Baseline**	**Follow-up**
	
Total BPRS	34.8 (9.7)	14.3 (8.2)**	26.9 (8)	23 (7.9)
Withdrawal - retardation	6.6 (4.0)	1.8 (2.2)**	3.2 (3.1)	2.9 (2.6)
Thinking disorder	4.4 (4.6)	1.3 (1.8)	1.7 (2.5)	1.8 (2.9)
Anxiety - depression	14.2 (4.4)	7.5 (3.7)**	14.0 (2.6)	11.6 (3.2)
Hostility - suspicious	4.3 (3.0)	1.5 (2.2)**	3.4 (3.3)	3.8 (2.3)
Activation	5.4 (3.7)	2.3 (2.9)*	4.6 (2.9)	2.9 (1.9)

*P ≤ 0.05; **P ≤ 0.001.

### BSI

There was no significant difference in the BSI total score between the control group (*M *= 84.1) and the experimental group (*M *= 74.2) at follow-up taking into account the covariate gender F = 1.031, *P *= 0.314, partial Eta^2 ^= 0.018.

### Client Observation

The mixed models ANOVA for the staff-rated Client Observation total found the Box's M test of equality of the covariance were significant with a higher mean score for the experimental group (*M *= 19.66) than the control group (M = 12.85) at baseline, thus the pretreatment Client Observation total score and gender were used as covariates. There was a significant difference between the groups at follow-up with greater improvement in the experimental group (M = 7.0, F = 11.859, *P *= 0.001, partial Eta^2 ^= 0.180).

Since there was a significant improvement in the staff-rated Client Observation total score of the experimental group, each of the items were examined using the mixed models ANOVA with the presubscale score and gender as covariates. The items were examined to look for differences in the different types of behaviors measured. All items, except for 'misperceptions of reality' (F = 3.704, *P *= 0.06, partial Eta^2 ^= 0.064), had significant improvement in the experimental group at follow-up (see Table 2). (Repetitively self-defeating behavior (F = 7.397, *P *= 0.009, partial Eta^2 ^= 0.120), self-absorbed or entranced (F = 11.440, *P *= 0.001, partial Eta^2 ^= 0.175), ever shifting priorities (F = 20.927, *P ≤ *0.001, partial Eta^2 ^= 0.279), and is needy, with ever shifting wants (F = 14.98, *P *≤ 0.001, partial Eta^2 ^= 0.217).) The items 'ever shifting priorities' and 'is needy, with ever shifting wants' had the largest effect sizes, suggesting they contributed the most to the change in the staff-rated Client Observation scale in the experimental group.

### Client Self-Observation

The mixed models ANOVA for the client self-report total score found the Box's test of equality of the covariance matrices and Mauchly's test of sphericity were not significant, therefore, assumptions were met. There was a significant difference in prescores and gender between the experimental and control groups, thus prescore and gender were used as covariates. There was a significant difference between the groups at follow-up with greater improvement in the total score of the client self-report in the experimental group (F = 6.246, *P *= 0.016, partial Eta^2 ^= 0.104). The items were examined to look for differences in the types of emotional states measured (see Table 3). Two of the items, mentally overloaded, overwhelmed (F = 6.037, *P *= 0.017, partial Eta^2 ^= 0.101) and circular rumination (F = 4.081, *P *= 0.048, partial Eta^2 ^= 0.07), were found to have greater improvement with a medium effect size in the experimental group than the control group at follow-up.

### Medication patterns

On admission to the crisis service there was no significant difference (*P *= 0.26) in the number of people who had stopped taking their medicine (control 42% n = 11, experimental 25% n = 8). However, there was a significant difference (*P *≤ 0.001) between the groups in the medication prescribing patterns. Medication prescribing patterns were divided into two groups, (a) those who had no change from the preadmission usual medication and dosage including restarting medication at previous dose, and (b) those who had their medication and/or dose changed. In the experimental group, 59% did not have changes made to their original medications. Changes to the medication regimen occurred more frequently in the control group, 92% versus only 41% of the experimental group.

There was a significant difference (*P *= 0.01) in the number of drugs between the control (M = 3.7) and experimental (M = 1.6) groups.

## Discussion

The results from the BPRS and from Client Observation by staff support the hypothesis that the experimental intervention would provide broad reduction of symptoms, as compared to treatment as usual. The finding was significant (*P *≤ 0.001) for total BPRS and four of five subscales, 'withdrawal/retardation', 'anxiety/depression' and 'hostility/suspicious', also (*P *≤ 0.05) for 'activation'. Similarly, the finding was significant (*P *≤ 0.001) for total Client Observation and for four of five items, 'self-absorbed/entranced', 'ever shifting priorities' and 'needy, with ever shifting wants', also (*P *≤ 0.05) for 'repetitively self-defeating behavior'. The results from the BSI show no significant improvement for either condition. From Client Self-Observation, the total score and scores for two of its nine items, 'mentally overloaded/overwhelmed' and 'circular rumination', are in favor of the experimental intervention (*P *≤ 0.05). The BSI and Client Self-Observation were the two instruments that used retrospective ratings for behavior before treatment. The experimental subjects received significantly fewer psychotropic medicines than the controls (*P *= 0.01).

### Patterns of symptom improvement

Aside from providing evidence for improvement among symptoms, the two pilot instruments, Client Observation and Client Self-Observation, were designed to obtain ratings for symptoms of interest, more specific for complex PTSD and BPD. As intended, the results from this study provide guidance for the further development of these pilot instruments.

According to the theory of the Cape Cod Model, the core dysfunction consists of repetitively regressive testing of someone's trustworthiness. The items 'repetitively self-defeating behavior' in Client Observation and 'mental overload', 'circular rumination' and 'inability to make judgments of trust' in Client Self-Observation depict that overall state of mind. Other symptoms, for example, 'self-absorbed/entranced', 'ever shifting priorities', 'hallucinations' and 'helplessness/depression', derive from the core dysfunction and they should surge or subside with it. Therefore, success of the experimental intervention should result in improvement across the board. However, treatment as usual, if presumed symptomatic, should result in uneven improvement only with continual effort. It should target behavior that is most burdensome to the patient or others (for example, hallucinations, urges to cut, neediness).

For the experimental group, Client Observation measured broad improvement, for four of five items, 'self-absorbed/entranced', 'ever shifting priorities' and 'needy, with ever shifting wants' (*P *≤ 0.001), also for 'repetitively self-defeating behavior' (*P *≤ 0.05). Among nine Client Self-Observation items, experimental subjects showed significant improvement for two of the three 'core' items, 'mental overload' and 'circular rumination' (*P *≤ 0.05). Improvement for the control group did not reach significance for any item of either scale.

Both groups gave themselves high pretreatment scores for the single most specific item, 'inability to make judgments of trust' (Table 3). This finding indicates that the control subjects did recognize the prevalence of that item in their mental operations retrospectively, when they were cued by the research raters, although presumably they had not been led to discover it during treatment, as the subjects in the experimental condition had. The possibility, however, that control subjects were suggestible to the raters' cues must be explored in the future.

### The place for medication

The results corroborate the prevailing understanding that medication mitigates certain symptoms and the reparative treatment of these disorders is good psychotherapy [41-43]. Subjects in the control group had more medication changes (*P *≤ 0.001) and received a larger number of drugs (*P *= 0.01) than experimental subjects. The efficacy of medication is best for quick reduction of excessive negative emotions and impulsivity, among all symptoms. It coincides with the timeframe of this study, 8 to 24 h of treatment. The hypothesized reparative intervention for the experimental group resulted in broad improvement, as noted above, compared to no significant improvement for the control group, even for negative emotions and impulsivity, in that time.

### The interface of therapy and the natural course of crises

Behavioral crises eventually subside in their natural course, without treatment. It is of interest to know how that factor may have contributed to the results from the experimental or the control group. Successful crisis interventions of different kinds must work either by enhancing the natural course or by making patients' behavior effective in a different way.

The theory guiding the experimental intervention explains behavioral crises as response to entrapment in treacherous intimacy in the present; then, it resolves them with correction of the hypothesized mechanism that compounds the entrapment while adhering to the goal of safe intimacy. By the same theory, treatment as usual should also shorten the duration of crises, however, by helping patients forego intimacy in the foreseeable future. Such is the natural closure of behavioral crises. Treatment as usual expedites it with symptom modulation and redirection, that is, reinvestment in evident priorities for non-intimate relationships. It creates conditions conducive to rethinking the futility of regressive testing and to letting go of the troubled opportunity for intimacy at hand.

With this understanding, ending a crisis with the experimental intervention has a cumulative value, beyond greater reduction of symptoms. It treats crises as stepwise lessons in management of the risks of intimacy and as the patient's introduction to more methodical lessons later, in anticipation of crises. To assess that cumulative value of therapy, future studies should measure grades of self-sufficiency in managing crises of trust without therapy.

### Lessons from the lifelong natural course

In addition to lessons from study of psychotherapy outcomes, there are good lessons to learn from studying the lifelong natural course of BPD and PTSD, that is, with little and unmethodical or no treatment [79]. One lesson that emerges resembles the concept that guides the experimental intervention, namely that it is possible for patients to seize opportunities for intimacy safely from the beginning of therapy. A second lesson is that doing so may be also necessary for therapy.

So far, the stepwise outcome with psychotherapy of different kinds has been remarkably parallel to that without treatment, but with a different pace. The typical natural course of these disorders leads to lesser frequency and intensity of crises, though with lasting avoidance of intimacy and emptiness [79-86]. Psychotherapy brings about a similar reduction of crises [47,58,75] seven times sooner [60]. Eventually, it labors with a similarly lasting avoidance of intimacy and emptiness [64,76,77]. But, then, in a few striking exceptions, sufferers without treatment somehow grow confident in intimate relationships, as someone's mother, brother or lifemate, and stay free of symptoms [33,80,87]. And, just as in the natural course, a few patients somehow take leaps of competence in particular relationships that cannot be attributed to progress in therapy [80].

A host of findings taken together begin to discern the forces at the fork, where the course of a few cases parts from the majority [5,87-93]. The emerging picture is that, with or without therapy, sufferers learn to preempt crises by avoiding experiments with intimacy, apparently from growing resignation. Those in therapy learn to avoid opportunities for intimacy faster than they learn to seize them safely with help from therapy [20]. But, for a few, either without or outside therapy, somehow someone helps them manage the dangers of love effectively, giving them stepwise, on the job lessons safely, without disorder. Learning the method of these natural healing agents should be instructive for psychotherapy [94].

### A spectrum of post traumatic disorders

The typical behavioral crises of complex PTSD and BPD resemble crises of dissociative identity disorder (DID), although the DSM-IV Text Revision (DSM-IV-TR) [95] omits that description from the criteria for DID. A recent line of inquiry entertains the notion of a spectrum of chronic post traumatic disorders comprising complex PTSD and BPD, also DID [9-13,96,97]. The inquiry is about identifying an essential mechanism that makes them all more alike than different. If that hypothesis is correct, one might extrapolate the results of this study to treatment for DID crises as well [98,99].

## Conclusions

The evidence presented in favor of the experimental intervention indicates that measurable in-depth improvement is possible even with treatment of a single crisis. If further studies prove this true, the outlook of crisis intervention will change, from palliation in the intervals of reparative psychotherapy to opportunity for in-depth reparation in its own right.

The challenge following this study is to ascertain that the broad reduction of symptoms demonstrated here ensues from the singular improvement that distinguishes the experimental intervention from other schools of treatment. Of course, the BPRS and the BSI do not measure repetition compulsion as such, nor do the instruments used in the cited studies capture the variously hypothesized core dysfunction in the operations of intimacy. Instruments must be developed to isolate the effect that each school of psychotherapy proposes differently as 'necessary and/or sufficient... [for] therapeutic progress' [76].

Furthermore, the pivotal effect of each therapy must be measured when it matters (that is, while patients are torn between need and fear in intimate relationships that define their future, unable to prove them safe and unable to imagine better ones). To date, outcome studies show that lessons from therapy's laboratories of intimacy, such as reworking old betrayals, reframing beliefs and analysis of the transference, do not generalize sufficiently to make intimacy in the social mainstream safe [62,64,76,77,94].

Another domain where the nature of the pivotal therapeutic intervention could be captured is the natural course of BPD and PTSD of DID. There are lessons to learn in studying how people with these disorders salvage few opportunities for intimate relationships compared to the many opportunities that they forego or that end in disorder [66,73]. Research could discern what makes the difference, whether the characteristics of patients or of their partners, skills and motives; then, therapy could learn to cultivate the necessary and sufficient ingredients directly in a patient's troubled relationships, in opportune time.

## Competing interests

The author declares that he has no competing interests.

## References

[B1] Bagge CL Stepp SD Trull TJ Borderline personality disorder features and utilization of treatment over two years J Personal Disord 2005 19 420 439 10.1521/pedi.2005.19.4.420 16178683

[B2] Gunderson JG Borderline Personality Disorder: A Clinical Guide 2001 Washington, DC, USA: American Psychiatric Press

[B3] Geller JL In again, out again: evaluation of a state hospital's "worst" recidivists Hosp Community Psychiatry 1986 37 386 390 2870978 10.1176/ps.37.4.386

[B4] Skodol AE Gunderson JG Pfohl B Widiger TA Livesley WJ Siever LJ The borderline diagnosis, I: psychopathology, comorbidity and personality structure Biol Psychiatry 2002 51 936 950 10.1016/S0006-3223(02)01324-0 12062877

[B5] Soloff PH Fabio A Prospective predictors of suicide attempts in borderline personality disorder at one, two and two-to-five year follow-up J Personal Disord 2008 22 123 134 10.1521/pedi.2008.22.2.123 18419233

[B6] Laddis A Dextraze A Fellman R The Cape Cod Model of psychotherapy In Proceedings of the American Psychiatric Association. 51st Institute of Psychiatric Services: Course 7 October 30, 1999; New Orleans, LA, USA

[B7] Herman JL Trauma and Recovery: The Aftermath of Violence From Domestic Abuse to Political Terror 1997 New York, USA: Basic Books

[B8] Freyd JJ Betrayal trauma: traumatic amnesia as an adaptive response to childhood abuse Ethics Behav 1994 4 307 329 10.1207/s15327019eb0404_1

[B9] Bremner JD Does Stress Damage the Brain: Understanding Trauma-Related Disorders from a Mind-body Perspective 2002 New York, USA: W. W. Norton & Company

[B10] Jung KE Posttraumatic spectrum disorder: a radical revision Psychiatric Times 2001 18 No 11

[B11] Laddis A Dell PF Dissociation and Personality Traits in 100 Persons With Borderline Personality Disorder In Proceedings of the VIII ISSPD Congress: October 10, 2003; Florence, Italy

[B12] Lonie I Borderline disorder and post-traumatic stress disorder: an equivalence? Aust N Z J Psychiatry 1993 27 233 245 10.3109/00048679309075772 8363532

[B13] Thorpe M Is borderline personality disorder a post-traumatic stress disorder of early childhood? Can J Psychiatry 1993 38 367 368 8348481 10.1177/070674379303800521

[B14] Yen S Shea MT Recent developments in research of trauma and personality disorders Curr Psychiatry Rep 2001 3 52 58 10.1007/s11920-001-0073-3 11177760

[B15] van der Kolk BA Roth S Pelcovitz D Sunday S Spinazzola J Disorders of extreme stress: the empirical foundation of a complex adaptation to trauma J Trauma Stress 2005 18 389 399 10.1002/jts.20047 16281237

[B16] Gunderson JG Sabo AN The phenomenological and conceptual interface between borderline personality disorder and PTSD Am J Psychiatry 1993 150 19 27 8417576 10.1176/ajp.150.1.19

[B17] Ford JD Courtois CA Courtois CA, Ford JD Defining and understanding complex trauma and complex traumatic stress disorders Treating Complex Traumatic Stress Disorders. An Evidence-Based Guide 2009 New York, USA: The Guilford Press 13 30

[B18] Classen C Pain C Field NP Woods P Posttraumatic personality disorder: A reformulation of complex posttraumatic stress disorder and borderline personality disorder Psychiatr Clin North Am 2006 29 87 112 10.1016/j.psc.2005.11.001 16530588

[B19] World Health Organization International Statistical Classification of Diseases and Related Health Problems: 10th Revision (ICD-10) 2007 2 Geneva, Switzerland: World Health Organization

[B20] Laddis A Regressive social learning results in chronic posttraumatic disorder ISSTD News 2008 26 5 7

[B21] Freud S Beyond the Pleasure Principle 1975 New York, USA: W. W. Norton & Company

[B22] Kitron DG Repetition compulsion and self-psychology: towards a reconciliation Int J Psychoanal 2003 84 427 441 10.1516/PHT8-9K7N-ELYY-G90F 12856360

[B23] Lazar R Repetition, repetition compulsion, motivation, interpretation Isr J Psychiatry Relat Sci 1998 35 9 19 9615526

[B24] Levy MS A conceptualization of the repetition compulsion Psychiatry 2000 63 45 53 10855759 10.1080/00332747.2000.11024893

[B25] Ponsi M Interaction and transference Int J Psychoanal 1997 78 243 263 9152753

[B26] Teicholz JG Kriegman D eds Trauma, Repetition & Affect Regulation: The Works of Paul Russell 1998 New York, USA: The Other Press

[B27] Kolk BA van der The compulsion to repeat the trauma. Re-enactment, revictimization, and masochism Psychiatr Clin North Am 1989 12 389 411 2664732

[B28] Bellino S Paradiso E Bogetto F Mood stabilizers and novel antipsychotics in the treatment of borderline personality disorder Psychiatric Times 2006 XXIII No 8

[B29] Triebwasser J Siever LJ Pharmacology of personality disorders Psychiatric Times 2006 XXIII No 8

[B30] Tyrer P Bateman AW Drug treatment for personality disorders Adv Psychiatr Treat 2004 10 389 398 10.1192/apt.10.5.389

[B31] Links PS Boggild A Sarin N Psychopharmacology of personality disorders: review and emerging issues Curr Psychiatry Rep 2001 3 70 76 10.1007/s11920-001-0076-0 11177763

[B32] Ahearn EP Krohn A Connor KM Davidson JR Pharmacologic treatment of posttraumatic stress disorder: a focus on antipsychotic use Ann Clin Psychiatry 2003 15 193 201 14971865 10.1023/b:acli.0000008173.01153.4e

[B33] Paris J Recent advances in the treatment of borderline personality disorder Can J Psychiatry 2005 50 435 441 16127960 10.1177/070674370505000802

[B34] Ipser J Seedat S Stein DJ Pharmacotherapy for post-traumatic stress disorder - a systematic review and meta-analysis S Afr Med J 2006 96 1088 1096 17164942

[B35] Grootens KP Verkes RJ Emerging evidence for the use of atypical antipsychotics in borderline personality disorder Pharmacopsychiatry 2005 38 20 23 10.1055/s-2005-837767 15706462

[B36] Friedman MJ Cohen JA Foa EB Keane TM Foa EB, Keane TM, Friedman MJ, Cohen JA Integration and Summary Effective Treatments for PTSD: Practice Guidelines from the International Society for Traumatic Stress Studies 2009 2 New York, USA: The Guilford Press 617 642

[B37] Friedman MJ Davidson JRT Stein DJ Foa EB, Keane TM, Friedman MJ, Cohen JA Psychopharmacotherapy for Adults Effective Treatments for PTSD: Practice Guidelines from the International Society for Traumatic Stress Studies 2009 2 New York, USA: The Guilford Press 245 268

[B38] Institute of Medicine Treatment of Posttraumatic Stress Disorder: An Assessment of the Evidence 2008 Washington, DC, USA: National Academic Press

[B39] Opler LA Grennan MS Ford JD Courtois CA, Ford JD Pharmacotherapy Treating Complex Traumatic Stress Disorders: An Evidence-Based Guide 2009 New York, USA: The Guilford Press 329 350

[B40] Raskind MA Shiromani PJ, Keane TM, LeDoux JE Pharmacologic treatment of PTSD Post-Traumatic Stress Disorder: Basic Science and Clinical Practice 2009 New York, USA: Humana Press 337 362

[B41] Oldham JM Guideline watch: practice guideline for the treatment of patients with borderline personality disorder Focus 2005 3 396 400

[B42] American Psychiatric Association Practice guideline for the treatment of patients with borderline personality disorder Am J Psychiatry 2001 158 Suppl 10 1 52 11665545

[B43] American Psychiatric Association Practice guideline for the treatment of patients with acute stress disorder and posttraumatic stress disorder Am J Psychiatry 2004 161 Suppl 11 3 31 15617511

[B44] Soloff P Special feature: psychobiologic perspectives on treatment of personality disorders J Personal Disord 1997 11 336 344 10.1521/pedi.1997.11.4.336 9484695

[B45] Bateman A Fonagy P Treatment of borderline personality disorder with psychoanalytically oriented partial hospitalization: an 18-month follow-up Am J Psychiatry 2001 158 36 42 10.1176/appi.ajp.158.1.36 11136631

[B46] Bateman A Fonagy P 8-year follow-up of patients treated for borderline personality disorder: mentalization-based treatment versus treatment as usual Am J Psychiatry 2008 165 631 638 10.1176/appi.ajp.2007.07040636 18347003

[B47] Clarkin JF Levy KN Lenzenweger MF Kernberg OF Evaluating three treatments for borderline personality disorder: a multiwave study Am J Psychiatry 2007 164 922 928 10.1176/appi.ajp.164.6.922 17541052

[B48] Giesen-Bloo J van Dyck R Spinhoven P van Tilburg W Dirksen C van Asselt T Kremers I Nadort M Arntz A Outpatient psychotherapy for borderline personality disorder: randomized trial of schema-focused therapy vs transference-focused psychotherapy Arch Gen Psychiatry 2006 63 649 658 10.1001/archpsyc.63.6.649 16754838

[B49] Linehan MM Tutek DA Heard HL Armstrong HE Interpersonal outcome of cognitive behavioral treatment for chronically suicidal borderline patients Am J Psychiatry 1994 151 1771 1776 7977884 10.1176/ajp.151.12.1771

[B50] Zanarini MC Frankenburg FR A preliminary, randomized trial of psychoeducation for women with borderline personality disorder J Personal Disord 2008 22 284 290 10.1521/pedi.2008.22.3.284 18540800

[B51] Zayfert C Deviva JC Becker CB Pike JL Gillock KL Hayes SA Exposure utilization and completion of cognitive behavioral therapy for PTSD in a "real world" clinical practice J Trauma Stress 2005 18 637 645 10.1002/jts.20072 16382429

[B52] Bateman AW Tryer P Psychological treatment for personality disorders Adv Psychiatr Treat 2004 10 378 388 10.1192/apt.10.5.378

[B53] Leichsenring F Leibing E The effectiveness of psychodynamic therapy and cognitive behavior therapy in the treatment of personality disorders: a meta-analysis Am J Psychiatry 2003 160 1223 1232 10.1176/appi.ajp.160.7.1223 12832233

[B54] Caligor E Psychodynamic treatments Psychiatric Times 2006 XXIII No 8

[B55] Rizvi SL Linehan MM Dialectical behavior therapy for personality disorders Focus 2005 3 489 494 10.1007/s11920-001-0075-1 11177762

[B56] Bateman AW Fonagy P Effectiveness of psychotherapeutic treatment of personality disorder Br J Psychiatry 2000 177 138 143 10.1192/bjp.177.2.138 11026953

[B57] Binks CA Fenton M McCarthy L Lee T Adams CE Duggan C Psychological therapies for people with borderline personality disorder Cochrane Database Syst Rev 2006 1 CD005653 16437534 10.1002/14651858.CD005652

[B58] Bradley R Greene J Russ E Dutra L Westen D A multidimensional meta-analysis of psychotherapy for PTSD Am J Psychiatry 2005 162 214 227 10.1176/appi.ajp.162.2.214 15677582

[B59] Cahill SP Foa EB PTSD: treatment efficacy and future directions Psychiatric Times 2007 24 No 3

[B60] Perry JC Banon E Ianni F Effectiveness of psychotherapy for personality disorders Am J Psychiatry 1999 156 1312 1321 10484939 10.1176/ajp.156.9.1312

[B61] Zanarini MC Frankenburg FR Hennen J Silk KR The longitudinal course of borderline psychopathology: 6-year prospective follow-up of the phenomenology of borderline personality disorder Am J Psychiatry 2003 160 274 283 10.1176/appi.ajp.160.2.274 12562573

[B62] Zanarini MC Frankenburg FR Reich DB Silk KR Hudson JI McSweeney LB The subsyndromal phenomenology of borderline personality disorder: a 10-year follow-up study Am J Psychiatry 2007 164 929 935 10.1176/appi.ajp.164.6.929 17541053

[B63] Linehan MM Behavioral treatments of suicidal behaviors. Definitional obfuscation and treatment outcomes Ann N Y Acad Sci 1997 836 302 328 10.1111/j.1749-6632.1997.tb52367.x 9616806

[B64] Levy KN Psychotherapies and lasting change Am J Psychiatry 2008 165 556 559 10.1176/appi.ajp.2008.08020299 18450934

[B65] Yehuda R Managing aggressive behavior associated with posttraumatic stress disorder J Clin Psychiatry Monograph 1999 17 25 27

[B66] Barley WD Buie SE Peterson EW Hollingsworth AS Griva M Hickerson SC Lawson JE Bailey BJ Development of an inpatient cognitive-behavioral treatment program for borderline personality disorder J Personal Disord 1993 7 232 240

[B67] McQuillan A Nicastro R Guenot F Girard M Lissner C Ferrero FL Intensive dialectical behavior therapy for outpatients with borderline personality disorder who are in crisis Psychiatr Serv 2005 56 193 197 10.1176/appi.ps.56.2.193 15703347

[B68] Ross C Acute stabilization and three-month follow-up in a trauma program J Trauma Dissoc 2004 5 103 112 10.1300/J229v05n01_06

[B69] Dubin SE Ananth J Bajwa-Goldsmith B Stuller S Lewis C Miller M Noel N Fernandez L Three day crisis resolution unit Indian J Psychiatry 1990 32 30 34 PMC2989565 21927423

[B70] Despland JN Drapeau M de Roten Y A naturalistic study of the effectiveness of a four-session format: the brief psychodynamic intervention Brief Treat Crisis Interv 2005 5 368 378 10.1093/brief-treatment/mhi026

[B71] Bloom BL Focused single-session psychotherapy: a review of the clinical and research literature Brief Treat Crisis Interv 2001 1 75 86 10.1093/brief-treatment/1.1.75

[B72] Winston AP Recent developments in borderline personality disorder Adv Psychiatric Treat 2000 6 211 217 10.1192/apt.6.3.211

[B73] Perimutter RA The borderline patient in the emergency department: an approach to evaluation and management Psychiatr Q 1982 54 190 197 10.1007/BF01064762 7182859

[B74] Slaby AE Trujillo M Psychotherapy and the suicidal patient Primary Psychiatry 2006 13 41 42

[B75] de Groot ER Verheul R Trijsburg RW An integrative perspective on psychotherapeutic treatments for borderline personality disorder J Personal Disord 2008 22 332 352 10.1521/pedi.2008.22.4.332 18684048

[B76] Linehan MM Special feature: theory and treatment development and evaluation: reflections on Benjamin's "models for treatment" J Personal Disord 1997 11 325 335 10.1521/pedi.1997.11.4.325 9484694

[B77] Benjamin LS Special feature: personality disorders: models for treatment and strategies for treatment development J Personal Disord 1997 11 307 324 10.1521/pedi.1997.11.4.307 9484693

[B78] Knowlton L Marsha Linehan: dialectic behavior therapy Psychiatric Times 1999 16 No 7

[B79] Paris J Personality Disorders Over Time: Precursors, Course, and Outcome 2003 Washington, DC, USA: American Psychiatric Press 10.1521/pedi.17.6.479.25360 14744074

[B80] Stone MH Long-term outcome in personality disorders Br J Psychiatry 1993 162 299 313 10.1192/bjp.162.3.299 8453424

[B81] Rosowsky E Gurian B Impact of borderline personality disorder in late life on systems of care Hosp Community Psychiatry 1992 43 386 389 1577432 10.1176/ps.43.4.386

[B82] Perkonigg A Pfister H Stein MB Höfler M Lieb R Maercker A Wittchen HU Longitudinal course of posttraumatic stress disorder and posttraumatic stress disorder symptoms in a community sample of adolescents and young adults Am J Psychiatry 2005 162 1320 1327 10.1176/appi.ajp.162.7.1320 15994715

[B83] Abrams R Sadavoy J, Lazarus LW, Jarvik LF Anxiety and personality disorders Comprehensive Review of Geriatric Psychiatry 1991 Washington, DC, USA: American Psychiatric Press 377 385

[B84] Paris J Borderline personality disorder CMAJ 2005 172 1579 1583 15939918 10.1503/cmaj.045281 PMC558173

[B85] Stevenson J Meares R Comerford A Diminished impulsivity in older patients with borderline personality disorder Am J Psychiatry 2003 160 165 166 10.1176/appi.ajp.160.1.165 12505816

[B86] Stepp S Pilconis PA Age-related differences in individual DSM criteria for borderline personality disorder J Personal Disord 2008 22 427 432 10.1521/pedi.2008.22.4.427 PMC2819124 18684054

[B87] Zanarini MC Frankenburg FR Hennen J Reich DB Silk KR Psychosocial functioning of borderline patients and axis II comparison subjects followed prospectively for six years J Personal Disord 2005 19 19 29 10.1521/pedi.19.1.19.62178 15899718

[B88] Gunderson JG Daversa MT Grilo CM McGlashan TH Zanarini MC Shea MT Skodol AE Yen S Sanislow CA Bender DS Dyck IR Morey LC Stout RL Predictors of 2-year outcome for patients with borderline personality disorder Am J Psychiatry 2006 163 822 826 10.1176/appi.ajp.163.5.822 16648322

[B89] Moran M Interpersonal relationships predict course of BPD Psychiatr News 2006 41 24

[B90] Paris J Implications of long-term outcome research for the management of patients with borderline personality disorder Harv Rev Psychiatry 2002 10 315 323 10.1080/10673220216229 12485978

[B91] Bhar SS Brown GK Beck AT Dysfunctional beliefs and psychopathology in borderline personality disorder J Personal Disord 2008 22 165 177 10.1521/pedi.2008.22.2.165 18419236

[B92] Luborsky L Popp C Luborsky E Mark D The core conflictual relationship theme Psychotherapy Research 1994 4 172 183 10.1080/10503309412331334012

[B93] Robins CJ Chapman AL Dialectical behavior therapy: current status, recent developments, and future directions J Personal Disord 2004 18 73 89 10.1521/pedi.18.1.73.32771 15061345

[B94] Laddis A Lessons from the natural course of chronic posttraumatic disorders25th Annual Conference of the International Society for the Study of Trauma and Dissociation, November 17, 2008, Chicago, IL

[B95] American Psychiatric Association Diagnostic and Statistical Manual of Mental Disorders DSM-IV-TR. (Text Revision) (IV-TR ed) 2000 Fourth Washington, DC, USA: American Psychiatric Association

[B96] Howell EF Blizard RA Dell PF, O'Neil JA Chronic relational trauma disorder: a new diagnostic scheme for borderline personality and the spectrum of dissociative disorders Dissociation and the Dissociative Disorders: DSM-V and Beyond 2009 New York, USA: Routledge

[B97] Strauss KL Differential diagnosis of battered women through psychological testing: personality disorder or post-traumatic stress disorder? Diss Abstr Int Sec B Sci Eng 1996 57 2166

[B98] Ishikura R Tashiro N Frustration and fulfillment of needs in dissociative and conversion disorders Psychiatry Clin Neurosci 2002 56 381 390 10.1046/j.1440-1819.2002.01026.x 12109955

[B99] International Society for Study of Dissociation Guidelines for treating dissociative identity disorder in adults J Trauma Dissoc 2005 6 69 149 10.1300/J229v06n02_07 16537324

